# Association of Sarcopenic Obesity and Osteoporosis in Postmenopausal Women: Risk Factors and Protective Effects of Hormonal Therapy and Nutritional Status

**DOI:** 10.1007/s11657-025-01573-w

**Published:** 2025-06-26

**Authors:** Oracha Chucherd, Orawin Vallibhakara, Sakda Arj-Ong Vallibhakara, Areepan Sophonsritsuk, Kitti Chattrakulchai, Makaramas Anantaburarana

**Affiliations:** 1 Reproductive Endocrinology and Infertility Unit, Department of Obstetrics and Gynecology, Faculty of Medicine, Ramathibodi Hospital, Mahidol University, Bangkok, Thailand; 2 Menopause Unit, Reproductive Endocrinology and Infertility Unit, Department of Obstetrics and Gynecology, Faculty of Medicine, Ramathibodi Hospital, Mahidol University, Bangkok, Thailand; 3 Interdisciplinary Studies and Lifelong Education, Faculty of Public Health, Mahidol University, Bangkok, Thailand

**Keywords:** Osteoporosis, Sarcopenia, Sarcopenic obesity, Menopause, Menopause hormone therapy, Nutritional status

## Abstract

***Summary*:**

The cross-sectional study of postmenopausal Thai women discovered a strong association between both sarcopenia and sarcopenic obesity and osteoporosis. The risk of sarcopenic obesity was found to increase with poor nutritional status, while a history of menopausal hormone therapy was shown to offer protection.

**Purpose:**

The study aims to investigate the association between sarcopenic obesity and osteoporosis in postmenopausal women and to identify risk factors for sarcopenic obesity.

**Methods:**

Our comprehensive cross-sectional study involved 248 Thai postmenopausal women aged 45-80. Osteoporosis was defined as a bone mineral density (BMD) T-score of less than −2.5 at the lumbar spine, total hip, or femoral neck, as measured by dual-energy X-ray absorptiometry (DXA). Sarcopenic obesity is defined as the co-existence of obesity and sarcopenia according to the criteria established by the European Society for Clinical Nutrition and Metabolism (ESPEN) and the European Association for the Study of Obesity (EASO). Sarcopenia was defined as skeletal muscle mass adjusted by weight (SMM/W) <35.6%, assessed via Bioelectrical Impedance Analysis (BIA), and compromised muscle function, which includes low hand grip strength (<18 kg) or poor physical performance (chair-stand test time ≥17 seconds). Obesity was defined as a fat mass percentage >41%, a body mass index (BMI) ≥25 kg/m^2^, or a waist circumference ≥80 cm. Moreover, a questionnaire of baseline characteristics and the factor associated with sarcopenic obesity was collected, including age, years since menopause, history of menopausal hormone therapy, underlying diseases, medications, nutritional status assessed by the Mini Nutritional Assessment (MNA), and physical activity assessed by The Global Physical Activity Questionnaire (GPAQ). Univariate and multiple logistic regression analyses were used to examine the associated factors with sarcopenic obesity.

**Results:**

The prevalence of sarcopenic obesity was 13.3%, and sarcopenia was present in 28.63%, while osteoporosis affected 39.91% of the participants. Sarcopenia and sarcopenic obesity were significantly associated with osteoporosis (odds ratio (OR) 3.05; 95% CI, 1.69-5.49; *p* < 0.05 and OR 2.65; 95% CI, 1.23-5.68; *p* < 0.05, respectively). In univariate and stepwise logistic regression analyses, a lower MNA score was significantly associated with an increased risk of sarcopenic obesity. Specifically, participants with an MNA score of 8–11 had an OR of 2.26; 95% CI,1.04-4.92; *p* < 0.04, while those with a score <8 exhibited a markedly elevated risk (OR 25.6; 95% CI, 1.04–4.92; *p* < 0.05). Conversely, the use of menopausal hormone therapy (MHT) was identified as a significant protective factor against sarcopenic obesity (OR 0.29; 95% CI, 0.10–0.79; *p* < 0.05).

**Conclusion:**

Both sarcopenia and sarcopenic obesity are linked to osteoporosis. Menopausal hormone therapy and nutritional status are significantly associated with sarcopenic obesity in postmenopausal women.

**Supplementary Information:**

The online version contains supplementary material available at 10.1007/s11657-025-01573-w.

## Introduction

Postmenopausal women, defined as those who have permanently ceased menstruation due to natural or surgical causes, experience significant hormonal changes, particularly a decline in estrogen, which leads to various menopausal symptoms, including vasomotor symptoms, mood disturbance, joint and muscle pain, and genitourinary syndrome of menopause [1, 2]. Moreover, the decline in estrogen levels significantly accelerates bone loss. This process begins around one year before menopause and continues for about three years afterward. Following this period, the rate of bone loss decreases slightly over the next four to eight years. On average, women lose approximately 10% of their bone mineral density (BMD) during the menopausal transition. These changes significantly increase the risk of osteoporosis, a skeletal disorder characterized by compromised bone strength and increased fracture risk. The pathophysiology involves accelerated bone remodeling where bone resorption exceeds formation due to estrogen deficiency, resulting in progressive bone loss [2, 3]. Osteoporosis affects approximately 20% of women in Thailand and constitutes a growing public health concern in the context of an aging population. The burden of osteoporotic fractures is considerable, with vertebral fractures being particularly problematic due to their often asymptomatic nature. Despite the absence of overt symptoms, these fractures are associated with increased morbidity and mortality. In Thailand, the prevalence of asymptomatic vertebral fractures among postmenopausal women has been reported to be as high as 29% [4, 5].

Sarcopenia, characterized by low muscle mass and function, frequently coexists with osteoporosis in older adults [6, 7]. The relationship between sarcopenia and osteoporosis is bidirectional, with sarcopenia contributing to an increased risk of osteoporosis through reduced mechanical loading on bones due to muscle loss, shared risk factors such as physical inactivity and chronic inflammation, and impaired muscle-bone crosstalk that disrupts osteogenic signaling pathways essential for bone formation [8]. In Thailand, sarcopenia affects 31.6% of women over 60, with higher rates in those over 70 or with a body mass index (BMI) < 18.5 kg/m2 [9].

Obesity, defined by excessive fat accumulation and typically measured by BMI, is another growing global health issue. It is associated with increased risks of cardiovascular disease, diabetes, and metabolic syndrome. The transition into menopause is often accompanied by weight gain and a shift toward abdominal and visceral fat, exacerbating these health risks. In Thailand, about 40% of postmenopausal women are classified as obese. The link between obesity and bone remodeling is not entirely understood. Some research suggests that obesity might positively affect bone density due to biochemical and hormonal interactions between fat tissue and bone. However, obesity can also cause low-grade inflammation, harming bone health and lowering bone mass [10–12].

Sarcopenic obesity, a condition that combines obesity and sarcopenia, affects around 10% of postmenopausal women, as indicated by a study in Taiwan [13]. This condition may occur due to either muscle loss leading to fat accumulation or obesity-induced muscle decline caused by metabolic issues. The interaction among fat, muscle, and bone tissue in sarcopenic obesity may contribute to changes in bone density and an increased risk of osteoporosis [14, 15]. This study aims to investigate the association between sarcopenic obesity and osteoporosis in postmenopausal Thai women. The study also aims to explore the risk factors associated with sarcopenic obesity and determine its prevalence within the study population.

## Method

### Study design

The cross-sectional study was conducted among postmenopausal women aged 45–80 who visited the outpatient menopausal clinic in Ramathibodi Hospital, Mahidol University, from November 2023 to April 2024. Eligible participants were required to be able to read and understand Thai and be willing to participate in the research by signing an informed consent form and completing the questionnaire. Data collection was performed after the ethical approval of the Ramathibodi Hospital Ethics Committee on Human Research, Faculty of Medicine, Ramathibodi Hospital (Number MURA 2023/752). We included postmenopausal women who visited a menopause clinic, had DXA data within a year, provided informed consent, completed the questionnaire, and underwent muscle strength testing and Bioelectrical Impedance Analysis (BIA) for muscle mass and fat mass.

We excluded women with other risk factors or diseases that could lead to osteoporosis, including those using steroid medications; those with endocrine disorders such as hyperparathyroidism or hyperthyroidism; those with gastrointestinal and malabsorption disorders, such as liver disease caused by excessive alcohol consumption, chronic inflammatory bowel disease, or vitamin D deficiency or insufficiency; those with bone marrow disorders such as leukemia or bone marrow cancer; those with a history of organ transplantation; and those with genetic disorders such as osteogenesis imperfecta. Additionally, we excluded patients with balance problems or difficulty sitting up or standing, and participants who did not complete the questionnaire.

The sample size was calculated using the two-independent proportion formula. The study's power was designed to be greater than 80%, with a P value below 0.05 considered statistically significant. Based on data from a previous Yen-Huai Lin et al. survey on the association of possible sarcopenic obesity with osteoporosis in postmenopausal women in Taiwan [13]. A detailed explanation of the sample size calculation, assumptions, and parameters is provided in the Supplementary Appendix.

## Definitions of sarcopenic obesity

According to the Joint Consensus Statement of the European Society for Clinical Nutrition and Metabolism (ESPEN) and the European Association for the Study of Obesity (EASO) [6], diagnosing sarcopenic obesity requires the co-existence of obesity and sarcopenia. Sarcopenia is identified based on low muscle mass and reduced muscle function, including strength and physical performance. Standard methods for measuring muscle mass are dual-energy X-ray absorptiometry (DXA) and Bioelectrical Impedance Analysis (BIA). This study used BIA with Tanita MC-780, Tanita Corporation, Tokyo, to assess muscle and fat mass. Sarcopenic obesity is defined as skeletal muscle mass adjusted by weight (SMM/W) < 35.6% and fat mass > 41% [6]. Sarcopenia is defined as Appendicular Skeletal Muscle Mass (ASM) < 5.7 kg/m2 according to the Asian Working Group for Sarcopenia (AWGS) 2019 Consensus [16]. Muscle strength is assessed using hand grip strength tests or chair-stand tests. The hand grip strength of the dominant hand was measured (in kilograms) using a dynamometer (Baseline® Hydraulic Hand Dynamometer, Fabrication Enterprises, White Plains, New York, USA). The participants were asked to sit with their elbows bent at a 90° angle for the hand grip strength measurement; the highest value out of three attempts was recorded. The diagnostic cut-off for low hand grip strength was < 18.0 kg for older Asian women [6]. Physical performance was assessed using the five-time sit-to-stand (5xSTS) test, which measures the time required for an individual to rise from a seated position to standing and return to sitting five consecutive times, without using their arms for assistance. Timing commenced when the examiner said “start” and concluded once the participant was seated after the fifth repetition. A chair-stand time of ≥17 seconds was considered indicative of poor performance for the diagnosis of sarcopenic obesity, while a threshold of ≥12 seconds was applied for sarcopenia alone [6, 16]. In this study, sarcopenia was defined as a skeletal muscle mass-to-weight ratio (SMM/W) <35.6%, measured using bioelectrical impedance analysis (BIA), in conjunction with compromised muscle function, defined by either low handgrip strength (<18 kg) or poor physical performance on the chair-stand test(≥17 seconds). Obesity was defined based on the Asia-Pacific guidelines issued by the World Health Organization (WHO) and the International Association for the Study of Obesity (IASO) for Asian populations. According to these criteria, obesity is diagnosed when body mass index (BMI) is ≥25 kg/m^2^, waist circumference exceeds 80 cm in women, or body fat percentage is greater than 41% [6, 11]. Sarcopenic obesity was defined as the coexistence of obesity, based on these Asian-specific criteria, and sarcopenia, as previously described.

## Diagnosis of osteoporosis

We included subjects who had undergone bone mineral density (BMD) measurements within the past year. The BMD measurement was carried out on the spine and hips of all participants following the official positions of the 2019 International Society for Clinical Densitometry (ISCD). The BMDs at the lumbar spine, total hip, and femoral neck were measured using DXA (Horizon W; Hologic Inc., Bedford, MA, USA). Osteoporosis was indicated by a T-score of ≤ −2.5 standard deviations at any site [2].

## Demographics, menopausal history, nutrition, and lifestyle

The participants'baseline characteristics were collected, including age, years since menopause, history of menopausal hormone therapy, underlying diseases, medications, nutritional status assessed by the Mini Nutritional Assessment (MNA), and physical activity assessed by The Global Physical Activity Questionnaire (GPAQ). Polypharmacy was defined as the use of five or more medications simultaneously, which has been associated with significant adverse health outcomes such as falls, cognitive impairment, and hospitalizations [17, 18].

The Mini Nutritional Assessment (MNA) is a screening and assessment tool used to evaluate nutritional status. It has a reliable scale with clearly defined thresholds. Healthcare professionals commonly employ the MNA in primary care settings to assess the nutritional status of elderly individuals living in the community due to its availability and user-friendliness. The MNA score, a 6-item questionnaire that includes: i) general clinical assessment (appetite and eating behavior, mobility status, psychological stress, neuropsychological problems), ii) physical measurements (weight loss and BMI), helps to identify normal nutritional status (MNA: 12–14), risk of malnutrition (MNA: 8–11), and malnutrition (MNA: 0–7) [19]. Physical activity includes any movement requiring muscle and energy, such as work, travel, or recreational activities. According to the World Health Organization (WHO), adequate physical activity entails at least 150 minutes of moderate physical activity per week, 75 minutes of vigorous activity per week, or a mix of both. The Global Physical Activity Questionnaire (GPAQ Version 2), translated into Thai, was employed to assess physical activity [20]. The GPAQ results were used to calculate weekly total energy expenditure, reported as metabolic equivalent minutes (MET) per week. The score is based on moderate or vigorous activities lasting at least 10 minutes, with the duration of moderate activity minutes per week multiplied by four and vigorous minutes multiplied by eight. Adequate physical activity was defined as a GPAQ score of at least 600 MET minutes per week, while insufficient physical activity was indicated by a GPAQ score below 600 MET minutes per week.

## Statistical analyses

The statistical analysis used STATA Version 16.0 (College Station, TX, USA). Descriptive statistics were calculated for participant demographics, anthropometrics, adipose tissue, muscle mass, and BMD. This included means and standard deviations for normally distributed variables and median and interquartile range for skewed variables. Factors associated with sarcopenic obesity were examined using univariate analysis using the Chi-square test. When a significant association of factors (p-value < 0.10) was identified, multiple logistic regression was employed to assess the independent effect of these factors. This study set statistical significance at p-value < 0.05, with a 95% confidence interval (CI). Individual age was treated as a continuous variable to ensure proper confounding control and included in all adjusted logistic regression models.

## Results

**Fig. 1:**
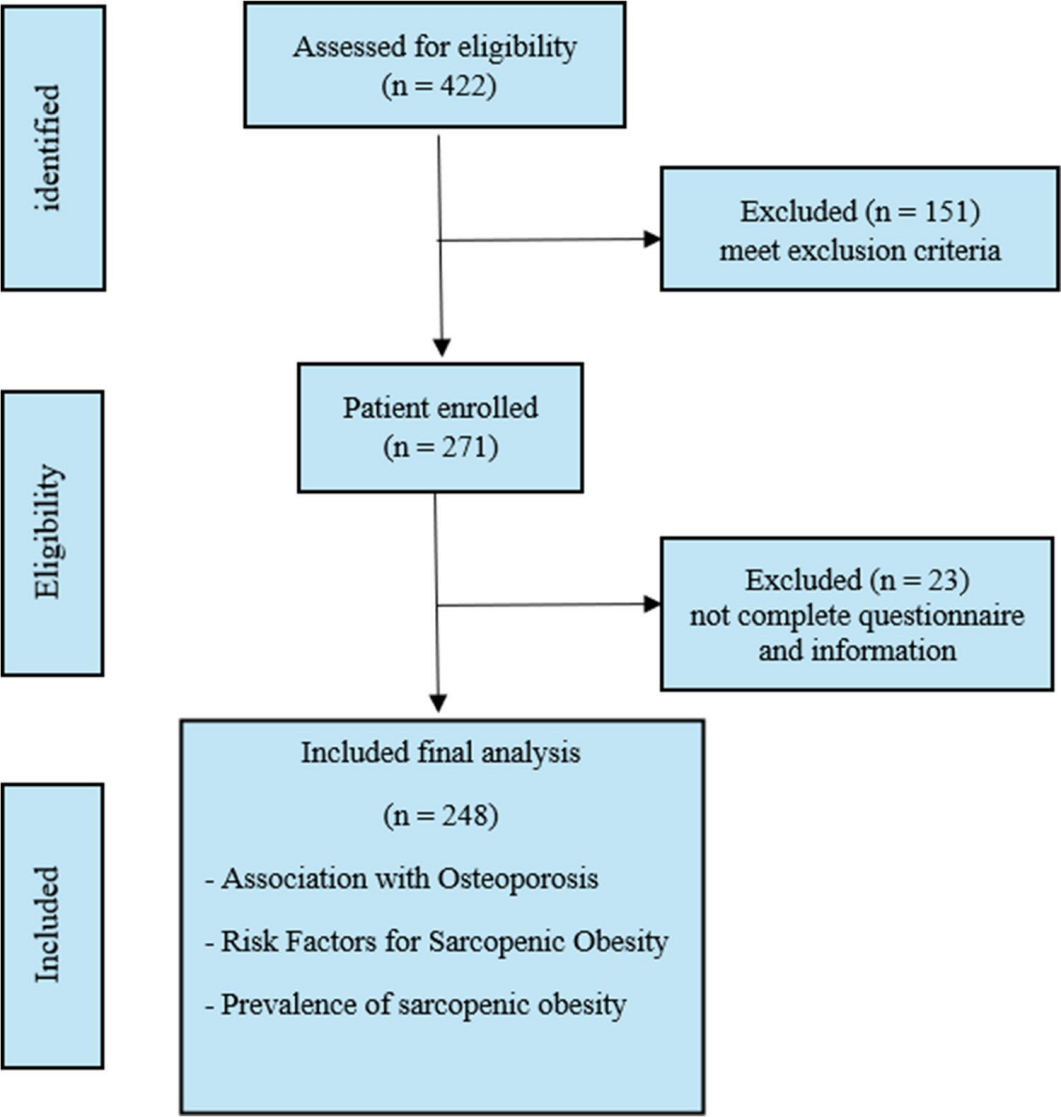
Study flow diagram

In our study, we included a total of 422 postmenopausal women. Of these, 248 women were enrolled for data analysis, as shown in the protocol flow chart. (Figure 1). The women had a median age of 67 (ranging from 45 to 80) and a median age of menopausal of 49 (ranging from 24 to 58), as shown in the baseline characteristics (Table 1). Most of the participants (89.52%) had underlying diseases, such as dyslipidemia, well-controlled hypertension, and diabetes Mellitus, indicating the complex health profile of this population. Additionally, most of the participants were retired (76.61%), physically active insufficient (72.58%), and had normal nutrition status (64.52%). Furthermore, 36.69% of the women had used menopausal replacement therapy, which was an average rate compared to the previous report in Thailand by Chaikittisilpa S et al. in 2007, which discovered that 43.1% of postmenopausal women were hormone users [21]. Some (17.4%) had experienced minor fractures, such as foot and forearm fractures. The mean BMI within the upper normal range was 22.8 (13.9 to 40.5) kg/m2. The waist circumference was 85 cm (ranging from 60 to 122 cm), slightly above the recommended cut-off of 80 cm for increased health risk in Asian women [22]. However, our study population had adequate muscle mass and function, with a median Appendicular Skeletal Muscle Mass (ASM) of 6.37 kg (ranging from 5.72 to 6.77 kg), median hand grip strength of 19.7 kg (ranging from 6.9 to 54 kg), and median Chair-stand test of 11 seconds (ranging from 4 to 23 seconds). In line with adequate bone health, the median Bone Mineral Density (BMD) in the lumbar spine, total hip, and femoral neck were 0.8085 g/cm^2^ (ranging from 0.49 to 1.37 g/cm^2^), 0.74 g/cm^2^ (ranging from 0.52 to 1.09 g/cm^2^), and 0.61 g/cm2 (ranging from 0.46 to 0.96 g/cm^2^), respectively, as shown in Table 2.

**Table 1:** Baseline characteristics of Participants

**Characteristics**	**n = 248**
Age, years*	67 (45–80)
Menopausal age, years*	49 (24–58)
Underlying Disease Yes No	222 (89.52) 26 (10.48)
History of Menopausal Hormone Therapy Yes No	91 (36.69) 157 (63.31)
Currently Employed Yes No	58 (23.39) 190 (76.61)
Education Below a bachelor's degree Bachelor Above a bachelor's degree	81 (32.66) 119 (47.98) 48 (19.35)
Parity Nulliparity One person More than one person	86 (34.68) 42 (16.94) 120 (47.98)
Alcohol Drinking Yes No	15 (6.05) 233 (93.95)
Polypharmacy ≥ 5 Yes No	205 (82.66) 43 (17.34)
Physical activity Adequate (GPAQ ≥ 600 MET-minutes) Insufficient (GPAQ < 600 MET-minutes)	68 (27.42) 180 (72.58)
Nutritional status Normal (MNA score ≥ 12) Risk of malnutrition (MNA score 8–11) Malnutrition (MNA score < 8)	160 (64.52) 84 (33.87) 4 (1.61)
Prior fracture (%) Yes No	44 (17.74) 204 (82.26)

Data is expressed as a number (%), *median, interquartile range (IQR),

GPAQ, Global Physical Activity Questionnaire; MNA, Mini Nutritional Assessment.

**Table 2:** Baseline characteristics associated with obesity, sarcopenia, and osteoporosis

**Characteristics**	**n = 248**
Body Mass Index, kg/m^2^	22.8 (13.9–40.5)
Waist circumference (cm)	85 (60–122)
Fat mass (kg) Fat percentage (%) Appendicular Skeletal Muscle Mass (kg/m^2^) Hand grip strength (kg)	14.60 (11.30–18.50) 26.95 (14.3–54.7) 6.37 (5.72–6.77) 19.7 (6.9–54)
Chair-stand test (second)	11 (4–23)
Basal metabolic rate (kcal)	1155.1 (960.0–1531.9)
Bone mineral density (BMD) (g/cm^2^) Lumbar Hip Femoral neck	0.8085 (0.49–1.37) 0.74 (0.52–1.09) 0.61 (0.46–0.96)
Sarcopenia*	71 (28.63%)
Obesity*	62 (25.0%)
Sarcopenic obesity*	33 (13.30%)
Osteoporosis*	99 (39.91%)

Data is expressed as *number (%), median, interquartile range (IQR)

In our study population, sarcopenic obesity was 13.3%, while the prevalence rates of obesity, sarcopenia, and osteoporosis were 25.0%, 28.63%, and 39.91%, respectively. Sarcopenic obesity was significantly associated with an increased risk of osteoporosis (OR 2.65; 95% CI, 1.23–5.68; *p* = 0.009). Notably, sarcopenia alone demonstrated the strongest association with osteoporosis (OR 3.05; 95% CI, 1.69–5.49; *p* < 0.05), whereas obesity alone was not significantly associated with osteoporosis (OR 0.76; 95% CI, 0.41–1.40; *p* = 0.37), as presented in Table 3.

**Table 3:** Association Between Sarcopenic Obesity, Obesity, and Sarcopenia With Risk of Osteoporosis

	Odds ratio	95% confidence interval	P-value
Sarcopenic obesity	2.65	1.23–5.68	0.009*
Obesity	0.76	0.41–1.40	0.37
Sarcopenia	3.05	1.69–5.49	0.0001*

* Statistically significant; p = <0.05

Univariate and multivariate logistic regression analyses identified nutritional status and the use of menopausal hormone therapy as independent factors associated with sarcopenic obesity, as shown in Tables 4 and 5. All adjusted models included chronological age as a covariate, treated as a continuous variable. Nutritional status, assessed using the Mini Nutritional Assessment (MNA), emerged as a significant determinant. Participants with an MNA score of 8–11 had significantly higher odds of sarcopenic obesity (OR 2.27; 95%CI, 1.05–4.91; *p* = 0.032). The risk increased markedly for those with an MNA score <8 (OR 9.67; 95% CI, 1.20–77.49; *p* = 0.032). These findings underscore the pivotal role of nutritional status in the development of sarcopenic obesity and suggest that nutrition-focused interventions may be essential in its prevention and management among postmenopausal women. In contrast, the use of menopausal hormone therapy was significantly associated with a reduced risk of sarcopenic obesity (OR 0.29; 95% CI, 0.10–0.79; *p* = 0.023), highlighting its potential protective effect on muscle mass and function during the postmenopausal period.

**Table 4:** Univariate Analysis of Potential Risk Factors for Sarcopenic Obesity

	Sarcopenic obesity n=33 N (%)	Control *n*=215 N (%)		Odds ratio	95% confidence interval	*p*-value
Age ≥ 60	29 (87.88)	166 (77.21)	2.14		0.71–6.43	0.16
Currently employed	4 (12.12)	54 (25.12)	0.41		0.14–1.23	0.10*
Polypharmacy	4 (12.12)	39 (18.14)	0.62		0.21–1.88	0.40
Underlying disease	27 (81.82)	195 (90.70)	0.46		0.17–1.26	0.12
History of MHT use	7 (21.21)	84 (39.07)	0.42		0.1–1.0	0.05*
Menopause age >45	13 (39.39)	72 (33.49)	1.29		0.61–2.75	0.51
History of fracture	4 (12.12)	40 (18.60)	0.60		0.20–1.82	0.36
Low grip strength (< 18 kg)	15 (45.45)	70 (32.56)	1.73		0.82–3.64	0.15
Chair-stand test (≥ 12 seconds)	13 (39.39)	88 (40.93)	0.94		0.44–1.99	0.87
High fat percentage (≥ 35%)	2 (6.06)	31 (14.42)	0.38		0.09–1.69	0.19
Risk of malnutrition (MNA score 8–11) Malnutrition (MNA score <8)	16 (48.48) 2 (6.06)	68 (31.63) 2 (0.93)	2.27 9.67		1.05–4.91 1.20–77.49	0.03* 0.01*
Adequate Physical activity	6 (18.18)	62 (28.84)	0.55		0.21–1.40	0.20

*Statistically significant; p ≤0.1

MHT, menopausal hormone therapy; MNA, Mini Nutritional Assessment

**Table 5:** Multivariate Logistic Regression Analysis of Independent Risk Factors for Sarcopenic Obesity

	Odds ratio	95% confidence interval	P-value
Currently employed	0.41	0.13–1.27	0.14
History of MHT use	0.29	0.10–0.79	0.023*
Risk of malnutrition (MNA score 8–11) Malnutrition (MNA score <8)	2.26 25.60	1.04–4.92 2.73–239.65	0.04* 0.004*

* Statistically significant; p = <0.05

MHT, menopausal hormone therapy; MNA, Mini Nutritional Assessment

## Discussion

This study provides robust evidence of a significant association between sarcopenic obesity and osteoporosis in postmenopausal women. Notably, sarcopenia alone conferred an even greater risk of osteoporosis compared to sarcopenic obesity, suggesting that the loss of muscle mass may be the primary driver of skeletal fragility. In contrast, obesity alone was not significantly associated with increased osteoporosis risk. These findings underscore that while the coexistence of muscle loss and excess adiposity, as seen in sarcopenic obesity, contributes meaningfully to osteoporosis risk, sarcopenia independently exerts the strongest influence.

Several potential mechanisms explain why sarcopenic obesity increases the risk of osteoporosis. First, sarcopenic obesity and osteoporosis are strongly associated with chronic low-grade inflammation, with pro-inflammatory cytokines such as IL-6, TNF-α, and IL-1 playing a critical role in developing both conditions. Second, lower levels of anabolic hormones, including growth hormones and sex hormones (estrogen, testosterone), contribute to muscle and bone loss as individuals age. Third, excess fat tissue in obesity releases adipokines that negatively affect muscle and bone metabolism, and increased oxidative stress in obesity can further harm muscle and bone tissue. Fourth, ectopic fat deposits in muscle and bone marrow directly harm muscle and bone. Finally, sarcopenic obesity often results in reduced physical activity, which negatively impacts both muscle and bone health [23–26]. Sarcopenia, rather than sarcopenic obesity, increases the risk of osteoporosis. This is due to several key factors. The loss of muscle mass and function in sarcopenia reduces bones'mechanical loading, which is crucial for bone formation. This reduced mechanical loading leads to decreased osteoblast activity and increased osteocyte apoptosis, negatively affecting bone health. Additionally, the muscle-bone crosstalk plays a vital role in maintaining bone health, with muscles functioning as an endocrine organ by secreting bioactive molecules known as myokines. Myokines such as irisin and osteocalcin promote bone formation and increase bone strength. In sarcopenia, the production of these myokines is reduced due to the loss of muscle mass, leading to impaired bone formation and an increased risk of osteoporosis. Furthermore, sarcopenia and osteoporosis share common causes, including hormonal changes, nutritional deficiencies, and physical inactivity, which increase the likelihood of their co-occurrence and exacerbate the risk of osteoporosis [27]. Obesity, intriguingly, can have both protective effects and potential risks concerning osteoporosis. On the beneficial side, the increased body weight in obese individuals leads to more significant mechanical loading on bones, resulting in higher BMD, which can lower the risk of osteoporosis [26]. Furthermore, the adipose tissue in obese individuals produces estrogen, a hormone known for its protective effect on bone, providing an additional advantage in bone preservation. [27, 28]. Our findings align with previous research reporting a significant association between sarcopenic obesity and osteoporosis in postmenopausal women. Antimo, Moretti, et al. (2014) [28] investigated 133 postmenopausal women and found that 35.3% had sarcopenic obesity, which was linked to elevated risks of both osteoporosis (OR 1.20; 95% CI, 0.58–2.50) and vertebral fragility fractures (OR 1.21; 95% CI, 0.56–2.62). Similarly, Chung et al. (2016) [26] demonstrated that sarcopenic obesity significantly increased the odds of osteoporosis (OR 2.93) in middle-aged and elderly Korean women. Supporting these findings, a meta-analysis by Gandham et al. (2021) [29], encompassing 31,540 older adults, revealed that individuals with sarcopenic obesity had lower femoral neck areal bone mineral density (BMD) compared to those with obesity alone, but higher BMD than those with sarcopenia alone. In contrast, Lin et al. (2022) [13] reported that potential sarcopenic obesity in postmenopausal Taiwanese women was paradoxically associated with a lower likelihood of osteoporosis (OR 0.28; 95% CI, 0.18–0.46), yet a higher risk of fragility fractures (OR 2.29; 95% CI, 1.31–4.00) and reduced trabecular bone scores, suggesting poorer bone quality despite preserved BMD. Similarly, Santos et al. (2018) [30] observed that in individuals aged ≥80 years, sarcopenia alone, rather than sarcopenic obesity, was more closely associated with lower BMD and increased osteoporosis risk Collectively, these studies highlight the critical importance of maintaining skeletal muscle mass to preserve bone health and reduce fracture risk, particularly in older and postmenopausal populations.

In our secondary analysis, several factors were identified as significantly associated with sarcopenic obesity in postmenopausal women. Multivariate logistic regression confirmed that menopausal hormone therapy (MHT) was associated with a reduced risk of sarcopenic obesity, whereas poor nutritional status remained an independent risk factor.MHT has been shown to preserve muscle mass and mitigate sarcopenia in postmenopausal women, counteracting the effects of menopause-related hormonal decline that contributes to both muscle loss and increased adiposity. Evidence from a large cohort study involving 4,254 postmenopausal women from the Korea National Health and Nutrition Examination Survey (KNHANES) demonstrated that prolonged MHT use was associated with higher muscle mass and a lower prevalence of sarcopenia, particularly among younger and leaner women [31]. The protective mechanisms of MHT are multifaceted. Estradiol has been shown to stimulate muscle satellite cells—muscle stem cells critical for regeneration and repair following injury or exercise. MHT also exerts anti-inflammatory effects, attenuating chronic low-grade inflammation contributing to muscle catabolism. Moreover, estradiol promotes an anabolic environment by enhancing muscle protein synthesis and reducing protein breakdown, which collectively supports muscle maintenance and growth [32–34].

In contrast, poor nutritional status is a key risk factor for sarcopenic obesity. Malnutrition and suboptimal diet quality can accelerate muscle wasting while promoting fat accumulation, particularly in visceral depots. This imbalance exacerbates physical decline and increases the risk of disability and other adverse health outcomes. Therefore, targeted nutritional strategies are crucial. Effective dietary management should emphasize adequate protein intake, including essential amino acids such as branched-chain amino acids (BCAAs), arginine, and glutamine, all known to stimulate muscle protein synthesis. These interventions can significantly improve muscle mass and functional outcomes when combined with resistance or strength-based exercise. A comprehensive, multidisciplinary approach that integrates hormonal therapy, nutritional optimization, and physical activity is essential for preventing and managing sarcopenic obesity in postmenopausal women [35–38].

Individuals with sarcopenic obesity are at an elevated risk of malnutrition compared to those without this condition. This dual burden creates a compounding effect, wherein sarcopenia is exacerbated despite preserved or even increased fat mass. Inadequate dietary quality and insufficient caloric intake are key contributors to progressive muscle atrophy alongside adipose tissue accumulation. A particularly critical factor is insufficient protein intake, common among malnourished older adults and directly associated with muscle mass, strength, and functional capacity declines. Malnutrition also promotes chronic systemic inflammation, accelerating muscle catabolism and enhancing visceral fat deposition. These interrelated mechanisms underscore the importance of comprehensive interventions targeting nutritional adequacy and metabolic balance. Addressing malnutrition in postmenopausal women with sarcopenic obesity is essential for interrupting this cycle and improving long-term musculoskeletal and metabolic health outcomes [39–41].

## Strengths and limitations

This study is strengthened by the use of well-established diagnostic criteria based on the 2022 consensus statement by the European Society for Clinical Nutrition and Metabolism (ESPEN) and the European Association for the Study of Obesity (EASO) [16], allowing for precise identification of sarcopenic obesity, sarcopenia, and osteoporosis in postmenopausal women. The study enhances its contextual relevance and applicability to the target population by applying Asian-specific reference cut-off values. Furthermore, the integration of validated assessment tools, including the Global Physical Activity Questionnaire (GPAQ) and the Mini Nutritional Assessment (MNA), enabled a comprehensive evaluation of key contributing factors such as physical activity and nutritional status. The deliberate exclusion of potential confounding variables further strengthens the internal validity and reliability of the findings.

However, several limitations should be acknowledged. First, the single-center design may limit the generalizability of the results. The study was conducted in a university hospital setting, so the sample may over-represent health-conscious individuals with higher socioeconomic status. Second, while the discussion considers the potential roles of hormonal regulation and inflammatory mediators (e.g., myokines and adipokines) in sarcopenic obesity and osteoporosis, these biomarkers were not directly measured. Third, although data on fracture history were collected, objective risk assessment tools such as the FRAX score and prospective fall-risk evaluation were not incorporated, potentially limiting the comprehensiveness of future fracture risk stratification.

Future research should consider multi-center, prospective designs that integrate biochemical markers, functional assessments, and validated fracture risk prediction tools to build a more holistic understanding of sarcopenic obesity and its skeletal implications in postmenopausal populations.

## Conclusion

Both sarcopenia and sarcopenic obesity are significantly associated with an increased risk of osteoporosis among postmenopausal women. Nutritional status and the use of menopausal hormone therapy emerged as key modifiable factors influencing the development of sarcopenic obesity. These findings underscore the importance of early identification and management of at-risk individuals through comprehensive strategies that address both muscle and bone health. Future research should prioritize longitudinal studies to establish causal relationships and assess the efficacy of targeted interventions.

## Supplementary Information

Below is the link to the electronic supplementary material.

Supplementary file1 (DOCX 25 KB)

## Data Availability

The data that support the findings of this study are not available due to ethical restrictions.
