# Supplementary material for: Association of Sarcopenic Obesity and Osteoporosis in Postmenopausal Women: Risk Factors and Protective Effects of Hormonal Therapy and Nutritional Status
Source: Arch Osteoporos. 2025 Jun 26;20(1):83. doi: 10.1007/s11657-025-01573-w (PMC12202630; doi:10.1007/s11657-025-01573-w)
Supplement: Supplementary file 1 — Supplementary file1 (DOCX 25 KB) [file 11657_2025_1573_MOESM1_ESM.docx]

**Supplementary Appendix: Sample Size Calculation Method**

The sample size was calculated using the formula for comparing two proportions, based on the study by Lin et al. (2022) on postmenopausal Taiwanese women, which reported an osteoporosis prevalence of 64% in the sarcopenic obesity group and 44% in the non-sarcopenic obesity group.

**Formula Used**


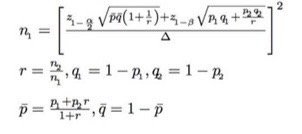


- alpha (α) = 0.05, beta(β) = 0.20
- p1 = 0.44, p2 = 0.64, Ratio(r) = 1
- Group 1 = 97, Group 2 = 97
- N = 194

Minimum required = ~194 participants total

To account for potential missing data and dropouts, we enrolled 248 participants.
